# An Electronic Structure Investigation of PEDOT with AlCl_4_^−^ Anions—A Promising Redox Combination for Energy Storage Applications

**DOI:** 10.3390/polym16101376

**Published:** 2024-05-11

**Authors:** Ben Craig, Peter Townsend, Carlos Ponce de Leon, Chris-Kriton Skylaris, Denis Kramer

**Affiliations:** 1 School of Engineering, University of Southampton, University Road, Southampton SO17 1BJ, UKc.a.ponce-de-leon-albarran@soton.ac.uk (C.P.d.L.); 2 Department of Chemistry and Chemical Biology, Rutgers University, 123 Bevier Rd., Piscataway, NJ 08854, USA; peter.townsend33@ntlworld.com; 3 School of Chemistry, University of Southampton, University Road, Southampton SO17 1BJ, UK; c.skylaris@soton.ac.uk; 4 Faculty of Mechanical Engineering, Helmut-Schmidt-University, Holstenhofweg 85, 22043 Hamburg, Germany

**Keywords:** PEDOT, AlCl_4_^−^, bipolaron model, electronic structure, conducting polymer, battery, aluminium

## Abstract

In this work, we use density functional theory to investigate the electronic structure of poly(3,4-ethylenedioxythiophene) (PEDOT) oligomers with co-located AlCl_4_^−^ anions, a promising combination for energy storage. The 1980s bipolaron model remains the dominant interpretation of the electronic structure of PEDOT despite recent theoretical progress that has provided new definitions of bipolarons and polarons. By considering the influence of oligomer length, oxidation or anion concentration and spin state, we find no evidence for many of the assertions of the 1980s bipolaron model and so further contribute to a new understanding. No self-localisation of positive charges in PEDOT is found, as predicted by the bipolaron model at the hybrid functional level. Instead, our results show distortions that exhibit a single or a double peak in bond length alternations and charge density. Either can occur at different oxidation or anion concentrations. Rather than representing bipolarons or polaron pairs in the original model, these are electron distributions driven by a range of factors. Distortions can span an arbitrary number of nearby anions. We also contribute a novel conductivity hypothesis. Conductivity in conducting polymers has been observed to reduce at anion concentrations above 0.5. We show that at high anion concentrations, the energy of the localised, non-bonding anionic orbitals approaches that of the system HOMO due to Coulombic repulsion between anions. We hypothesize that with nucleic motion in the macropolymer, these orbitals will interfere with the hopping of charge carriers between sites of similar energy, lowering conductivity.

## 1. Introduction

Despite having been used in a variety of applications, the fundamental understanding of conducting polymers (CPs) is still incomplete and remains a highly active area of research. CPs are semiconducting in their pure form, and only become conductive when an oxidising or reducing species co-locates with the polymer chain via a redox reaction, a process conventionally referred to as doping by analogy with doped inorganic semiconductors. CPs have a carbon backbone consisting of alternating single–double carbon bonds [1,2]. PEDOT continues to be the subject of particularly intensive research due to its chemical stability and tuneable conductivity [3]. To date, PEDOT has found applications in supercapacitors [4], sensors [5], solar cells, bioelectronics, electrochemical transistors, electrochromic displays [6], battery electrodes [7] and spintronics [8]. The present work studies the PEDOT/AlCl_4_^−^ system, inspired by the use of PEDOT as a cathode in a battery with AlCl_3_:EMIm[Cl] electrolyte and an aluminium anode. In this context, PEDOT achieves 1000 cycles and a theoretical maximum energy density of around 50–55 Wh kg^−1^ based on all active materials. Although this is less than the typical values achieved for Al-graphite, which is the state of the art for non-aqueous aluminium batteries, it still represents a promising avenue of research [9]. The co-locating of PEDOT with a small symmetrical anion also allows the comprehensive analysis of the electronic structure of PEDOT relevant to a wider field and is a useful complement to the more commonly studied larger anions such as polystyrene sulfonate (PSS) or tosylate (Tos) [1].

While many computational studies on PEDOT exist to date [10,11,12,13,14], to the best of our knowledge, the present study is the first computational study that concerns the introduction of AlCl_4_^−^ onto PEDOT, except for the authors’ own prior work [15,16].

### 1.1. Structure and Electronic Properties of PEDOT and the Bipolaron Model

In conventional semiconductors, the high coordination number (four and greater) of covalent bonds between atoms to their neighbours provides a rigid structure such that electronic excitations can be viewed as electrons or holes in a lattice. In CPs, each monomer unit is bound to a maximum of two other monomer units by carbon–carbon bonds, making CPs subject to structural distortions caused by the addition or removal of electrons. Much of the early theoretical work on conducting polymers focussed on attempting to fit existing semiconductor theory to these new materials.

**Figure 1:**
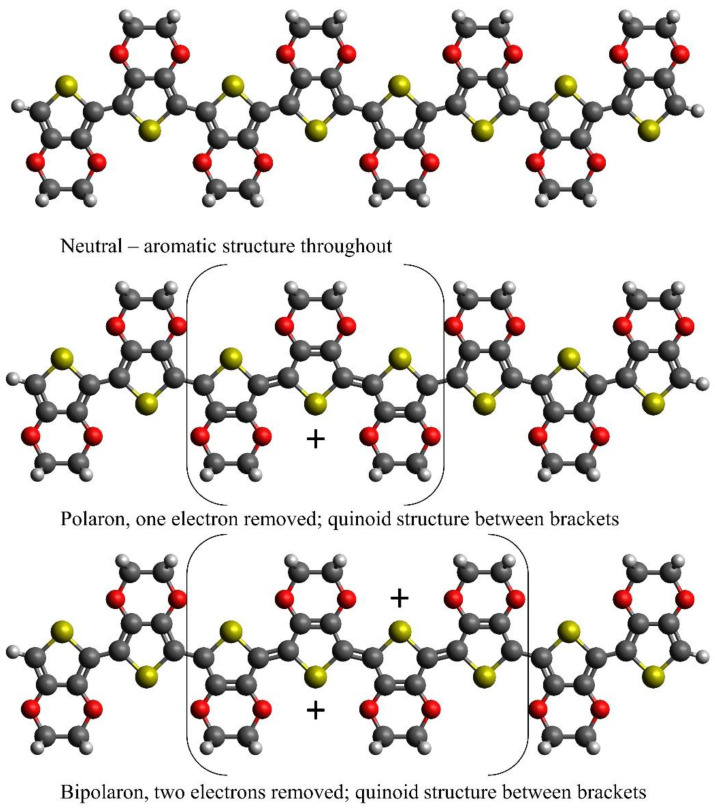
The 8-PEDOT, according to the original 1980s bipolaron model, demonstrating the proposed form of a polaron and bipolaron compared to a neutral oligomer, with accompanying inversions of the single–double carbon–carbon bonds. Yellow atoms are sulphur, red atoms are oxygen, grey are carbon and white are hydrogen. The positive charges are indicated on single carbon atoms with three single bonds but are distributed over a few monomer units as indicated by the brackets. The bond lengths are not to scale and are illustrative only. Adapted from the review by Heinze et al. [17].

It has been consistently observed and predicted that when counterions are added to conducting polymers, localized charge-carrying distortions occur over a length of a few monomer units [17,18] as shown in Figure 1. A key feature is that the change in bond lengths observed closely corresponds to the charge density. Electron kinetic energy drives increased distortion length, while the lattice relaxation drives decreased distortion length; the resulting length is a balance of both [14,19]. The theory of few-monomer distortion lengths is based on the observation that the distortions in conducting polymers are localized around the part of the chain where the anions are located, and their orbitals do not mix enough to form extended energy bands [20]. Instead, they form a series of localized, overlapping states with significant electronic and structural distortions [21]. The fact that the distortions are localized has led researchers to term them polarons.

The bipolaron model in its original form was developed from theoretical and experimental results in the 1980s, shortly after conducting polymers were discovered, in an attempt to explain some of their key characteristics. For example, in inorganic semiconductors, trapped polarons would be expected to build up throughout the lattice as they are oxidised. In contrast, when a conducting polymer is oxidised, the spins build up, before disappearing again, with no spins detected in the fully charged polymer [18,22,23]. To explain this, the bipolaron model was devised, which stated that the charges first appear as localised singly charged states termed polarons with half a spin, and then as the polymer is further oxidised, the charges co-locate into doubly charged spinless states termed bipolarons, explaining the disappearance of the spins [18]. Polarons and bipolarons according to the original 1980s bipolaron model are shown in Figure 1.

The single–double bond pattern in the conjugated backbone is critical to the characteristics of conducting polymers. For the simplest conducting polymer, trans-polyacetylene, the hydrogen atoms are on alternating sides of the carbon atoms. The conjugated carbon backbone has alternating single–double bonds due to a Peierls distortion [24]. No change in energy results from inverting the single–double bond pattern: that is to say, the two possible orientations are degenerate. The presence of a counterion introduces a local region with inverted single–double bond patterns, termed a soliton in the 1980s bipolaron model [25].

Thiophene derivatives including PEDOT have three carbon–carbon bonds in each monomer unit, and so bond inversion is not energetically neutral. A neutral PEDOT monomer unit has a long central bond and a shorter bond either side, described as an aromatic structure. The inverted pattern appears in the presence of anions; this is described as a quinoid [25,26], as shown in Figure 1. A polaron is the term given to a localized single-charged distortion, with an accompanying surrounding lattice distortion carrying a distinct charge, analogous to polarons found in inorganic polar insulators and semiconductors [25]. PEDOT and polythiophene are typically treated with anions, becoming oxidised (p-doped), so the polarons are hole polarons [27].

When the polymer is further oxidised, so that it carries two charges per oligomer, the bipolaron model predicts that the charges locate together into a doubly charged excitation termed a bipolaron [28], illustrated in Figure 1. The theoretical driving force is that the energy saved by structural relaxation is greater than the energy penalty from the Coulombic repulsion between the two like charges [14]. Further increases in anion concentration lead to multiple bipolaron formation [18]. The 1980s bipolaron model was supported by predictions from early theoretical methods such as the one-electron extended Hückel theory. For example, the Hückel theory finds that the energy of distortion to form two polarons or one bipolaron is quite similar, but the ionization energy is less for a bipolaron than two polarons [18]. The Hückel theory predicted polarons and bipolarons to be around four monomer units long in polypyrrole [29]. A semiempirical analysis using the modified neglect of diatomic overlap (MNDO) method showed similar results [30].

When conducting polymers are in their pure state and unoxidised, they have a bandgap characteristic of semiconductors and the HOMO–LUMO gap is too large to allow much or any conduction. The bipolaron model, set out by Brédas et al. [18] and based on an infinite periodic polymer with a band structure, predicts that the removal of an electron from a neutral conducting polymer causes the half-filled energy level to be pushed upwards into the gap. The half-filled level is termed the polaron level. An empty state also appears below the conduction band, termed the bipolaron level. The appearance of these states reduces the bandgap. Removing a further electron causes the newly unoccupied polaron level to be pushed up further still, with a similar decrease in the energy of the bipolaron level, further reducing the bandgap [18].

Brédas et al. also predicted that the valence band, not including the half-filled level which is described as the polaron band under the bipolaron model, would always be full [18]. It was proposed that the levels appearing in the bandgap corresponded to the π (bonding) and π* (antibonding) states created by the mixing of the anionic states with the existing system [29]. Regardless of debate over the bipolaron model, it is well known that the reduction in the bandgap via the introduction of anions is a key contributor to increased conductivity.

The actual mechanism of conductivity in conducting polymers is a mix of intra- and interchain electron and hole mobility. Conductivity along a PEDOT molecule occurs once there are sufficient numbers of well-distributed anions adsorbed onto it. This causes the quinoid regions to overlap and pass electrons or holes down the conjugated carbon backbone via resonance between many states of nearly identical energy, with many orders of magnitude larger conductivity than the pure polymer [21]. This is possible via the rapid swapping of the single–double bonds which allow the charge carriers to travel efficiently [5]. This mechanism means that conductivity along the PEDOT molecules is typically very high at moderate anion concentrations. This movement can be thought of as the intrachain hopping of charge carriers between nearby sites of similar energy [4].

The hopping or tunnelling of charge carriers between different PEDOT molecules and regions of the polymer is also required for conductivity. PEDOT and other conducting polymers are generally a mix of ordered and disordered regions with some degree of self-ordering due to *π*–*π* interactions. In the ordered regions, conductivity depends on the direction of hopping in the stacked polymer. Various models have been developed or adapted for interchain conductivity [4], including Mott’s variable range hopping (VRH) [31] or Sheng’s fluctuation-induced tunnelling [32]. Further work has suggested that only some regions in the PEDOT achieve polaronic structure and that hopping must also take place between these highly conductive regions [33]. In general, interchain hopping between different molecules or regions has been found to be the limiting factor in conductivity [4].

The bipolaron model predicted that as the concentration of counterions rises, the spinless bipolarons become mobile and are able to conduct due to the broadening of the bipolaron band, which forms from the bipolaron level as many electrons are removed [17]. At the highest counterion concentrations, the bands can even merge to produce a metal or semimetal [21]. This explanation fits well with the experimentally observed increase in the conductivity of conducting polymers at low anion concentrations.

However, experiments on conducting polymers including several thiophene derivatives, albeit not including PEDOT, have shown that conductivity increases up to counterion concentrations of 0.5, then decreases above this [34], for which the bipolaron model offers no explanation [17]. The same behaviour was observed for a hexathiophene and polyphenylene hybrid [35]. Heinze et al. suggested that conducting polymers have a maximum of conductivity at 0.5 due to the same mechanism as radical ion salts [17,36]. This is proposed to be the point at which there is a maximum of charge carriers available to hop between sites of similar energy on different chains. Heinze et al. proposed that during the wide plateau of conductivity, overlapping redox states are reached successively, keeping the system in a mixed valence state until the last redox sites are emptied and the conductivity drops [17]. Meanwhile, Brédas et al. attributed this discrepancy with the bipolaron model to the screening of the attraction between counterions and their polarons at high anion concentrations [18]. Others have found that at high anion concentrations, the anions can cluster into regions which inhibit electron hopping between PEDOT regions [37]. Ofer et al. suggested that the presence of both charged and uncharged sites would be required for charge carrier hopping. They theorized that the loss of conductivity at high anionic concentrations was due to some kind of localization of charges due to Coulombic repulsion between solvent and charges in the highly charged states [34].

The research in this area is limited partly because of a generally accepted view that anion concentrations normally reach a maximum of about 0.4 in electrochemically synthesized conducting polymer films [17,38,39]. Higher levels are achievable, but special electrolytes are required to reach concentrations above 0.5 [34]. No studies could be found that examined PEDOT conductivity specifically as a function of calculated anion concentrations above 0.5, but several show a trend for conductivity to drop as the concentration of anions available for adsorption increases past a certain point [40,41,42,43].

What all these mechanisms have in common is that they rely on charge carriers hopping between different sites of similar energy. In this work, it is found that at high anion concentrations of 0.66 on single PEDOT molecules, the Coulombic repulsion between the anions pushes up the energy of their highly localised orbitals so that they are close in energy to the HOMO of the overall system, which is normally a delocalised orbital extending over the conjugated carbon backbone. In this thesis, it is suggested that combined with the movements of the polymer at temperatures above 0 K, these highly localised orbitals might be pushed up in energy sufficiently that they begin to interfere with the hopping mechanism between different sites of similar energies, affecting both intra- and interchain conductivity.

### 1.2. Progress from Density Functional Theory

Since the 1980s, when the original bipolaron model was developed, DFT, particularly hybrid DFT incorporating a proportion of Hartree–Fock exact exchange energy, has become the standard approach for modelling conducting polymer systems due to its ability to handle electron correlation effects while remaining efficient for reasonably large systems [16]. Investigations via DFT have provided significantly different insights into the electronic structure of conducting polymers and have led to revised explanations of experimental observations.

A key limitation of one-electron theories is that they omit electron–electron repulsion [14]. One-electron methods assume the degeneracy of the spin orbitals even for spin-asymmetric configurations with a partially occupied level, treating the half-filled band as fully occupied. The spin degeneracy assumption distorts the results between odd and even oxidation levels, as the independence of spin-up (*α*) and spin-down (*β*) orbitals is an important part of the energy minimization process for any open-shell system. Consequently, the Hückel theory predicts that two new spin-degenerate levels are created in the gap for each oxidation step of the PEDOT; a lower singly occupied state and upper unoccupied state for a polaron and two empty states for a bipolaron [44]. In contrast, ab initio methods do not predict spin degeneracy for polarons, and only one state appears in the gap [6]. Meanwhile, semiempirical methods need parameterising carefully from appropriate ab initio methods [14].

Hybrid DFT calculations generally do not support the existence of the type of spinless bipolaron predicted by the one-electron or semiempirical approaches where charges co-locate through the energetic preference of structural relaxation [6,45]. A review of DFT studies indicates that bipolarons do not exist in long oligomers without counterions [14]. Only oligomers shorter than eight units long [14], and oligomers with one counterion per six or fewer thiophene rings [46] exhibit bipolaron-like behaviour [10], and then only because there is not enough room for the distortions to separate into polarons. Also, spectroscopic experiments on oligothiophenes have demonstrated that the sub-gap transitions previously attributed to bipolarons were actually two separated polarons in long oligothiophenes [47]. Findings such as this led to the invention of the term polaron pair, which consists of two polarons for which it is not energetically favourable to co-locate, so they tend to move apart along the chain if there is space to do so. In this conception, bipolarons are closed-shell singlet states and therefore spinless, while two polarons on the same chain can be singlet or triplet biradicals [48].

The presence of counterions also serves to pin charge carriers in a particular location, which can give rise to apparent bipolaronic behaviour, generating overlapping distortions that appear as one larger one [25]. The modelling of thiophene chains of various lengths up to 25 monomer units has indicated that polarons tend to spread out but do interact still in the middle of the chain, observed via bond length changes compared to neutral oligomers, implying an interaction spread over a much larger part of the chain. These results have also been interpreted to support the concept of polaron pairs rather than bipolarons, referring to the triplet state reported for oligomers longer than ten monomer units with +2 oxidation state which shows two peaks in bond length alternations [49].

In contrast to previous explanations of the build-up and disappearance of spins, more recent DFT work has proposed that the tendency for spins to build up during oxidation and then reduce or disappear once oxidation is complete is caused by the removal of multiple single electrons from fully occupied valence states across different chains in the polymer and then the subsequent removal of the singly occupied electrons, leaving the original HOMO-1 as the new, fully occupied HOMO [6]. The differences between the bipolaron model and the modern view are subtle, but they can be summarised as that the bipolaron model predicts the incorrect band structure and predicts that two like charges have a structural preference for co-location in oxidised conducting polymers with and without counterions. Modern DFT research unanimously rejects the band structure of the original bipolaron model and generally rejects the idea of a structural tendency for two like charges to co-locate in oxidized conducting polymers without anions, at least not at the ground state, although at higher multiplicities single, larger distortions can appear. The presence of nearby counterions in oxidised conducting polymers that are relatively fixed in position can also generate larger, combined distortions due to overlapping distortions.

To account for the developments from modern theoretical methods, it has become convention to refer to the original 1980s bipolaron model as the ‘traditional model’ or its predictions as those of the ‘traditional methods’. Since 2019, some authors have adapted the terms from the original bipolaron model to fit modern understanding [6,50]. Perhaps the clearest elucidation of the updated terms polaron, bipolaron and polaron pair is provided by Sahalianov et al., with a polaron referring to a doublet with +1 charge, a polaron pair to a triplet with +2 charge and a bipolaron to a singlet with +2 charge [50]. In this approach, states with more than two charges are referred to as polaron(ic) or bipolaron(ic) for odd and even charged states, respectively [6,50]. An important feature is that the terms are no longer linked explicitly to distortion characteristics, unlike less recent theoretical work [10,48,49]. Sahalianov et al. also updates the definition of a bipolaron to mean a distortion shared between two nearby anions and a polaron pair to mean two anions that are far enough apart to produce two separate distortions, but with no structural preference for the co-location of charges implied for either [50]. Ambiguity remains over how to describe what happens when more than two anions are near enough to share a single distortion.

This change in definition means that a bipolaron or a polaron pair is no longer associated with a distortion shape or to an energetic preference for co-location or separation of two charges, leaving very little of the original meaning. This change in terminology can easily be confusing to those not familiar with the latest theoretical work. This is evidenced by the fact that many researchers are still using the bipolaron model in its original 1980s form based on pre-DFT methods [17,21,27,39,51,52,53,54,55,56,57,58], as also pointed out elsewhere [6,50]. For clarity and because others still base their understanding on it, we adopt the convention of referring to the bipolaron model in its original 1980s form and describing our own results from first principles, and then clarifying the new definition of polarons and bipolarons in the Conclusion.

It is important to note that the investigation of oxidised conducting polymers without counterions is really a hypothetical study problem. In the battery context, and in other real-life contexts, it cannot be expected that pure PEDOT can be oxidised without counterions present to balance the charges. Despite this, conducting polymers without anions have been thoroughly investigated by now. Therefore, a more pertinent research question going forwards is to focus on the electronic structure of conducting polymers with counterions.

## 2. Methodology

DFT simulations were carried out on single chains of PEDOT in a vacuum, both without anions and also with different numbers and arrangements of anions. These simulations were analysed for energy levels, charge distribution, molecular orbitals and bond lengths. Initial PEDOT geometries were constructed via the creation of a single monomer unit followed by relaxation and then the repeated addition and relaxation of additional monomer units.

The lengths of the oligomers were chosen based on the estimate that PEDOT consists of molecules 5–20 monomer units in length [6,17,59,60].

In the calculations on oxidised PEDOT without counterions, the neutral relaxed geometry for each oligomer length was used as the starting geometry for all oxidised configurations, with deliberate asymmetries randomly introduced to check for symmetry pinning that might otherwise freeze the geometry in an unstable configuration. The appropriate number of electrons were then removed from the system and spin state was applied, and then relaxation was carried out to establish the relaxed geometry of the oxidised molecules. In all cases, the oxidised molecules relaxed to form symmetrical bond lengths, angles and charge distributions either side of the central carbon–carbon bond. The work on PEDOT oligomers without anions used Gaussian 09 [61]. These calculations were carried out to B3LYP 6-31+G* [62,63,64] with Grimme’s D3 dispersion corrections with Becke–Johnson damping [65].

Configurations with anions were assembled by finding a starting position for anions relative to sulphur atoms for which the DFT calculation reliably converged. We have shown in a previous work using ab initio molecular dynamics that this approach finds minima suitably close in energy to global minima [15]. The work on PEDOT oligomers with anions was completed using NWChem 6.8 [66], with B3LYP 6-31G* with D3 dispersion corrections for the geometry relaxations. We found that including diffuse functions in the basis set did not significantly change the results. Gaussian 09 [61] was subsequently used for the Merz–Kollman electrostatic potential (ESP) charge analysis of the relaxed structures, which was conducted using a custom point density of 5 points per unit area on 5 spherical layers [67].

## 3. Results and Discussion

In the results and discussion, firstly, the B3LYP and wB97XD functionals are compared on the basis of bond lengths and partial charges. Following this, a detailed discussion takes place of bond lengths, partial charges and energy level structures of different lengths of PEDOT oligomer with different numbers of anions. Bond lengths and partial charges are chosen for analysis because they are the parameters most affected by the formation of distortions. The energy level structures meanwhile allow comparisons to the band structures found via experimental measurements and to predicted band structures based on traditional methods. Finally, molecular orbitals show the character of different orbitals, enabling further comparison to predictions based on the traditional methods.

### 3.1. Functional Comparison

Historically, B3LYP has been used extensively for modelling conducting polymers [10,11,14,48,68,69,70,71]. However, recently, there has been some movement towards the range-separated hybrid functional wB97XD [6,12]. The main reason for moving away from B3LYP is due to its over-delocalization of electrons, with no localisation of distortions found in singly charged oligomers without anions. However, much of the research has focussed on polymers without anions. This is an unphysical situation as the polymers cannot be oxidised without counterions in experiment. The introduction of anions is known to pin distortions locally. Therefore, it was important to test whether wB97XD predicts different results from B3LYP for a number of test cases: a neutral 12-PEDOT without anions; 12-PEDOT at +1 and +2 oxidation states without anions; and 12-PEDOT with one and two anions.

**Figure 2:**
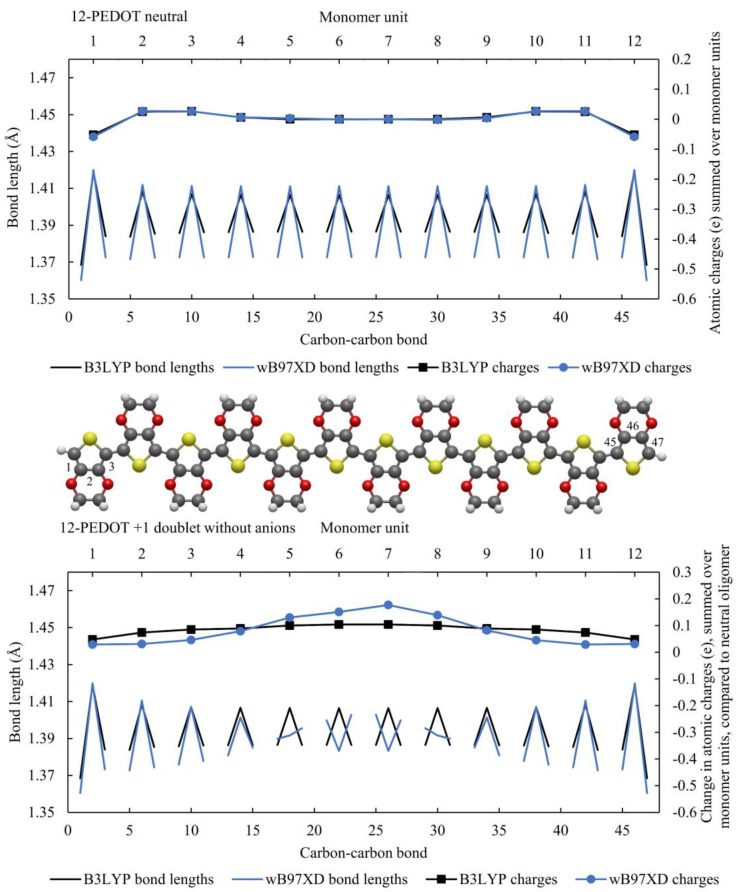
The 12-PEDOT modelled with B3LYP and wB97XD, showing bond lengths and summed charges on each monomer unit. For the neutral case (top), the charges are summed. For the 12-PEDOT +1 without anions, the charges are the difference in summed charges per monomer unit from the neutral oligomer. The carbon–carbon bonds between monomer units are negated in order to highlight the aromatic vs. quinoid structure. This means that bond lengths of the three carbon bonds within the monomer unit are displayed, creating an arrow shape pointing either upwards or downwards. The monomer units with the arrow shape pointing upwards have aromatic structure, while those with the arrow pointing downwards have quinoid structure.

Figure 2 shows the neutral 12-PEDOT oligomer and the 12-PEDOT +1 doublet without anions. The neutral case shows almost identical results in terms of charge distribution. In terms of quinoid vs. aromatic regions, where a monomer unit has aromatic structure if the bond lengths form an arrow shape pointing upwards and quinoid if the arrow shape points downwards, all monomer units have aromatic structure for both functionals. However, wB97XD predicts a greater difference between short and long bonds. For the neutral case then, there is qualitatively little difference between the two methods.

For the 12-PEDOT +1 doublet without anions, there is a pronounced difference between the two functionals. The bond lengths of wB97XD show a quinoid region localised over two monomer units with a sharp transition on the monomer units either side. In contrast, B3LYP predicts that the difference between short and long bond lengths slightly reduces. This is a key difference between the two functionals, with B3LYP tending towards a delocalisation of distortions along the whole oligomer in PEDOT without anions while wB97XD results in strongly localized distortions. This has also been shown elsewhere as previously discussed [12]. The charges follow the distortion location: wB97XD consequently has a charge peak around the middle of the oligomer. The slight asymmetry in summed charges is attributed to a rounding error in the partial charge calculation.

**Figure 3:**
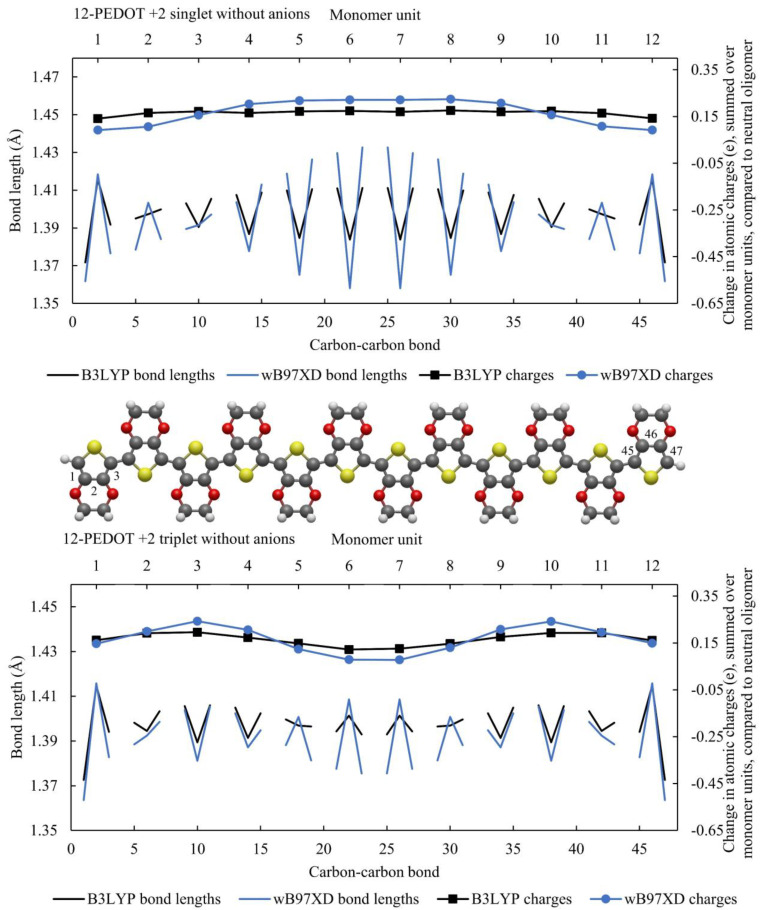
The 12-PEDOT +2 singlet and triplet modelled with B3LYP and wB97XD, showing bond lengths and summed charges on each monomer unit. The charges are the difference in summed charges per monomer unit compared to the neutral oligomer. The carbon–carbon bonds between monomer units are negated in order to highlight the aromatic vs. quinoid structure.

The results for 12-PEDOT +2 singlet and triplet without anions are shown in Figure 3. The results for each functional are much more similar for the singlet case. wB97XD results in a distortion that is one monomer unit shorter in length at each end, showing a quinoid region spanning six monomer units rather than the eight for B3LYP. Again, the charge profile for B3LYP is highly delocalised. In contrast, wB97XD results in a charge profile that has a wide plateau over the length of the distortion. These findings confirm a significant difference between the two functionals for PEDOT without anions, and given the known over-delocalisation of electrons resulting from B3LYP, suggests that wB97XD may offer more accurate insights for the anion-free case.

**Figure 4:**
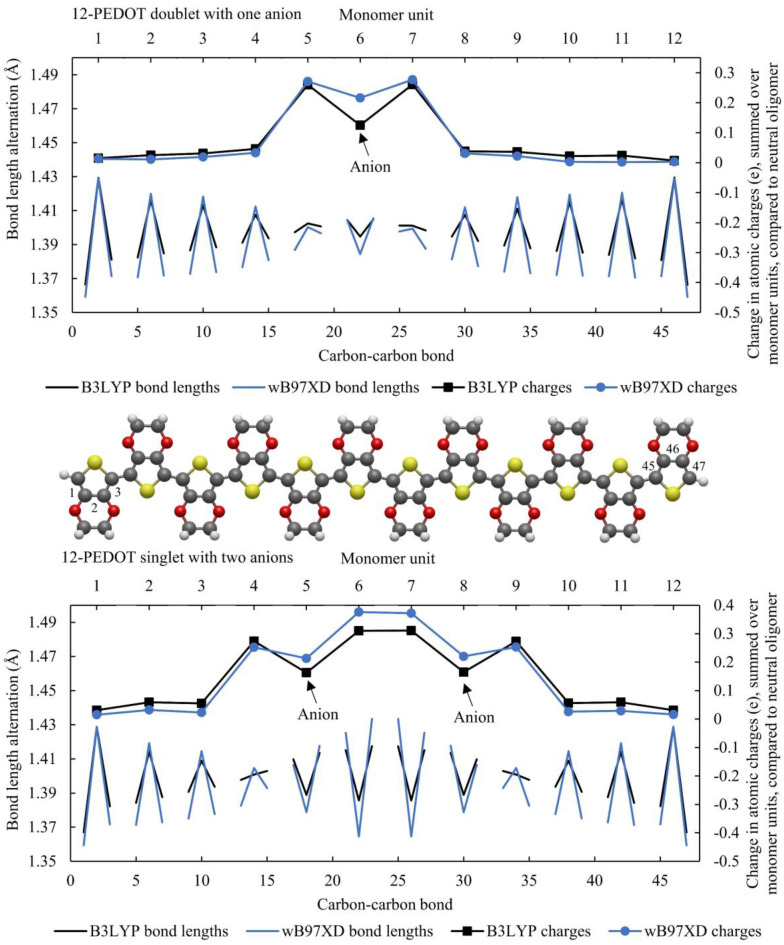
The 12-PEDOT with one and two anions modelled with B3LYP and wB97XD, showing bond lengths and summed charges on each monomer unit. The charges are the difference in summed charges per monomer unit compared to the neutral oligomer. The carbon–carbon bonds between monomer units are negated in order to highlight the aromatic vs. quinoid structure.

To analyse the system with anions, the 12-PEDOT with one anion (singlet ground state) and the 12-PEDOT with two anions (doublet ground state) are shown in Figure 4. In both cases, the areas of quinoid and aromatic structure are much more similar between the two functionals than for oxidised PEDOT without anions, with the only difference qualitatively being that in the case of the 12-PEDOT with two anions, wB97XD gives bondf lengths that transition from aromatic to quinoid between one monomer unit and the next, while B3LYP shows a transition monomer unit with a structure that is not clearly aromatic or quinoid. With anions, the summed charges per monomer unit relative to the neutral oligomer are also much more closely matched between functionals. It is a known error of the correction for the self-interaction error that DFT systematically underestimates charge transfer in redox reactions when this is known in reality to be almost always complete. Therefore, the dip in charge around the anions can be attributed to negative charge spilling back from the anion. wB97XD predicts a smaller error of this kind, shown by a smaller dip in the summed charge, but the error persists. These results show that B3LYP does not predict significantly more delocalized distortions when anions are present compared to wB97XD, suggesting that either functional can be used to produce similar results in this system.

While this work focusses on PEDOT with AlCl_4_^−^ anions, it is worth noting a few key observations from these graphs. Both functionals show a single peak distortion in bond lengths and charge distributions for the 12-PEDOT +2 singlet and a double peak distortion for the 12-PEDOT +2 triplet. Noting that the exact energy levels of the ground states are not very reliable from any DFT functional, it can be said that either the singlet or triplet state could exist for the 12-PEDOT +2 without anions. Furthermore, the peaks in the charge distribution do not add up to a double or two single charges as would be expected for a bipolaron or two separated polarons (a polaron pair). The same observation can be made from Figure 2 for 12-PEDOT +1. Instead, the charge is distributed over the full length of the oligomer, even for wB97XD. This directly contradicts the original bipolaron model which states that a polaron or bipolaron are linked to particular distortion characteristics, and in this case, both single and double peak distortions are possible for the same oxidation state.

### 3.2. Limits of Computational Approach and Choice of Functional

It is notable that delocalization error is one of the biggest remaining challenges to overcome with DFT and no existing functional offers a complete solution [72]. It is responsible for predicting incomplete charge transfer for redox pairs when unity charge transfer is expected as well as the aforementioned delocalization of electrons. Meanwhile, static correlation error is linked to inaccuracies in the estimations of the energies of degenerate states, which can sometimes be avoided by using broken symmetry calculations. This problem is linked to the fact that DFT uses a single Slater determinant: it is monodeterminantal. Multideterminantal methods are used in situations where this is a problem, but they are not possible for larger systems [73]. During calculations for the current work, attempted broken symmetry calculations always failed to converge and the issue is avoided by discussing only the qualitative electronic structure of the different low-lying excited states, rather than drawing firm conclusions about which is the true ground state.

In summary, while B3LYP and wB97XD predict quite different results for oxidised PEDOT without anions, the differences between them are small for neutral oligomers and when anions are introduced. Therefore, in the study of conducting polymer systems with anions, it is not evident that range-separated hybrid functionals provide an advantage, and therefore, B3LYP is used throughout the rest of this study.

### 3.3. Bond Length and Partial Charge Analysis

When AlCl_4_^−^ anions are introduced to the system, they locate in stable energy wells over the sulphur atoms and between the two oxygen atoms, out of the plane of the PEDOT oligomer, and can adsorb either side of the plane of the fused rings. Previous work has shown that this position is generally stable, providing only one anion is incorporated per PEDOT oligomer; however, if several anions are positioned on adjacent PEDOT oligomers, they may spread out to adjacent sites during DFT structural relaxation or ab initio molecular dynamics [15]. This result contrasts with the original bipolaron model which predicts an energetic preference for the co-location of charges.

To examine the effects of different numbers of anions on the chain, a 6-PEDOT and 12-PEDOT system were considered with a number of anions from zero to four, which could be placed anywhere except the end monomer units. Low energy configurations were chosen from the full range of possible configurations for 6-PEDOT and a large random sample of configurations for 12-PEDOT. The actual configurations used can be seen in the Supplementary Materials, Figure S1.

**Figure 5:**
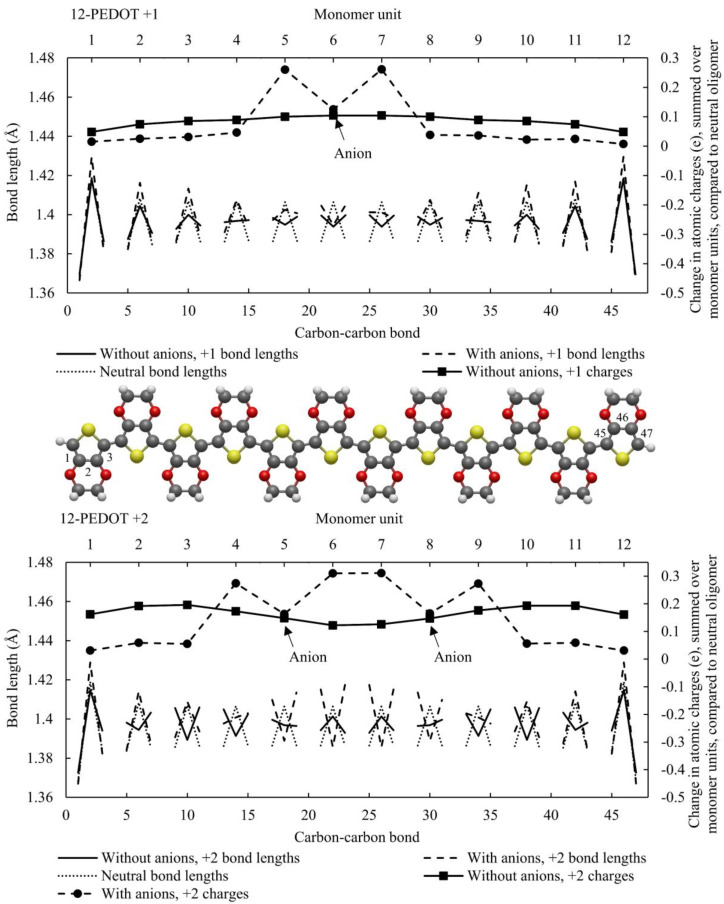
Changes in atomic partial charges calculated from the ESP summed over monomer units compared to the neutral oligomer, and bond lengths of the carbon–carbon bonds. Upper panel: changes in charge density compared to the neutral oligomer, and bond lengths for 12-PEDOT, both without counterions at +1 oxidation state, and with a single anion on monomer unit 6. The bond lengths of the neutral oligomer are shown for reference. The end three units remain sharply aromatic in both cases, as previously observed, indicating a continued tendency for negative charge to collect at the ends of the chain. The system has a doublet ground state. The anion causes the bond length alternation (and area of positive charge) to locate on a smaller region only three monomer units long, demonstrating how anions pin the distortions in place. Lower panel: 12-PEDOT +2 without counterions shows the triplet (ground) state, and with anions, the singlet (ground) state with two anions on monomer units 5 and 8. The end units remain sharply aromatic in both cases, indicating a continued tendency for negative charge to collect at the ends of the chain. The 12-PEDOT without counterions, which has a triplet ground state, demonstrates two separate distortions and charge peaks while the system with anions, which has singlet ground state, forces the excitation to locate on the four monomer units closest to the anions, with a peak of positive charge in this region.

Figure 5 shows the effect of introducing one or two anions to 12-PEDOT in terms of bond lengths and electron distribution. The configurations chosen are the lowest energy anion configurations from a large sample. When a single anion is introduced on monomer unit 6 (12-PEDOT +1), the bond length alternations localise over just the three monomer units nearest the anion, strongly localising the distortion. This result demonstrates that the introduction of anions results in highly localised distortions. For the +2 system with counterions, 12-PEDOT was set up with anions on monomer units 5 and 8. The charge profile and bond length alternations show a distortion that was six monomer units long, showing that where two anions are close together, a single distortion can occur that spans both.

As observed for 12-PEDOT without anions in Figure 3, there are two general forms of distortion that appear in the PEDOT systems studied in this work: a single or double peak distortion. It is found here that both can occur for PEDOT systems with anions as well. Figure 5 shows that for 12-PEDOT with anions, a single peak distortion occurs for one and two anions, noting that the charge density exhibits a dip on the monomer unit where the anion is located, with no accompanying change in the bond lengths. This is attributed to DFT incorrectly calculating the anions as having less than -1e charge. This single peak distortion observed is a similar result to that presented by Sahalianov et al. who found that two counterions a relatively short distance apart would cause a single peak distortion with singlet ground state, which they referred to as a bipolaron, whereas two anions further apart could induce a double peak distortion with triplet ground state, which they termed a polaron pair [50]. The possibility of a polaron pair in this definition is not explored here; to observe it, Sahalianov et al. used an 18-oligomer, longer than is explored in this work.

**Figure 6:**
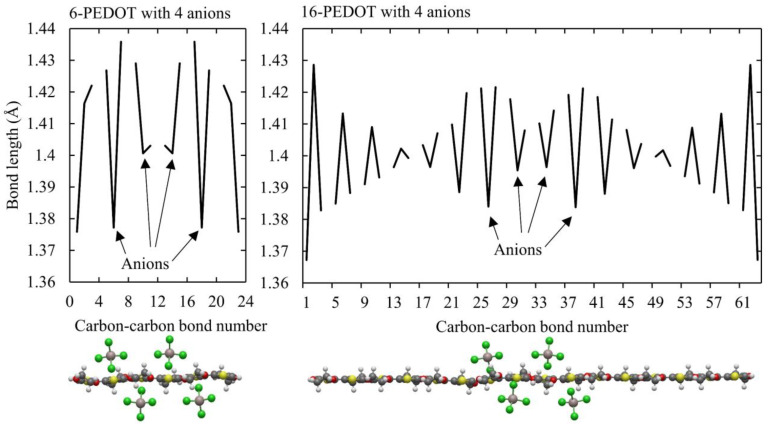
Bond lengths for 6- and 16-PEDOT with four anions. Left panel: bond lengths for 6-PEDOT with four anions on alternating sides of the central four monomer units, with the anion locations shown on the PEDOT oligomer below. A pronounced double peak region of quinoid structure is seen. Right panel: bond lengths for 16-PEDOT with four anions on alternating sides of the central four monomer units, with the anion locations shown on the PEDOT oligomer below. A pronounced double peak region of quinoid structure is also seen, although it is localised towards the middle of the oligomer due to the location of the anions. Below: the molecular structures of 6-PEDOT with four anions (left) and 16-PEDOT with four anions (right). For the anions, the light grey atoms are aluminium and the green atoms are chlorine. For the PEDOT, the yellow atoms are sulfur, the dark grey atoms are carbon, the red atoms are oxygen and the white atoms are hydrogen.

Figure 6 shows that for 6- and 16-PEDOT, when four anions are placed on adjacent monomer units on the oligomer to examine the effects of a high anion concentration on a localised part of a longer oligomer, a strong double peak distortion occurs. This implies that higher oxidation states are more associated with double peak distortions. In the case of 16-PEDOT with four anions in Figure 6, the pinning of the distortion around the anions is seen as the distortion only extends over eight monomer units.

### 3.4. Energy Level Structure of Neutral and Oxidised 6- and 12-PEDOT with Anions

**Figure 7:**
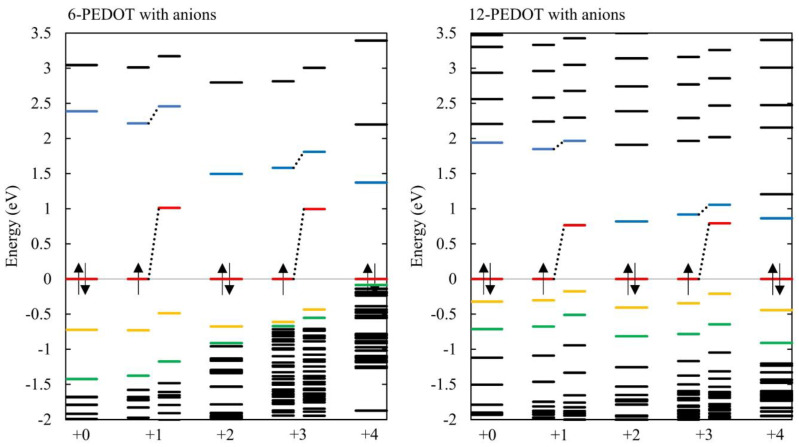
For filled valence shells the HOMO is indicated with both up- and down-spin electron arrows, while for partially filled valence shells only the partially filled α HOMO are indicated. The fully occupied or partially filled HOMOs are coloured red, the HOMO-1 are coloured yellow, and HOMO-2 are coloured green; the LUMO is coloured light blue. At high doping levels for 6-PEDOT, there is a close grouping of energy levels below the HOMO because the non-bonding orbitals of the anions have been introduced. These are heavily localised on the chlorine atoms of the anions (see Figure 9) and so can bunch closely as they do not interact much with each other. Otherwise, the plots with anions are qualitatively similar to those without counterions (Figure 8) with a general decrease in bandgap as electrons are progressively removed from the PEDOT oligomer. For 12-PEDOT compared to 6-PEDOT, the close clusters of energy levels occur further down the energy scale because for 12-PEDOT, the overall anion concentration is lower, and the anions are more spread out. The triplet ground state seen in Figure 8 for 12-PEDOT disappears for 12-PEDOT with two anions, which has singlet and doublet ground states throughout. The configurations are shown in Supplementary Materials, Figure S1. The HOMOs are normalized to 0 eV as for Figure 8.

**Figure 8:**
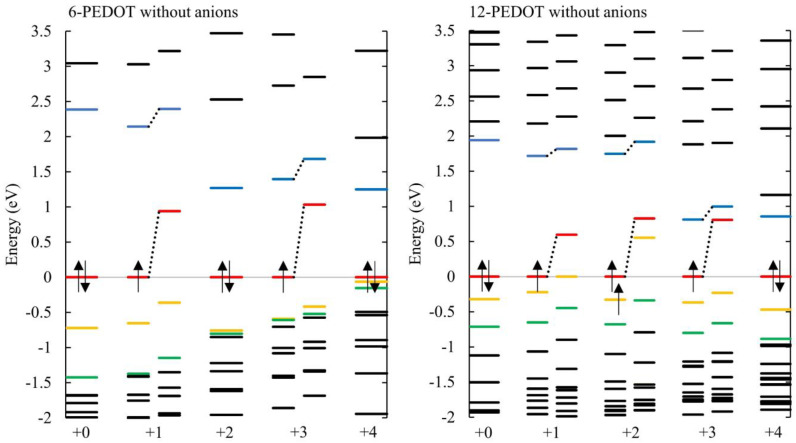
Energy levels for 6-PEDOT and 12-PEDOT, without counterions, at different oxidation states. It is shown that for odd charges, with doublet ground state, a single unoccupied layer appears in the gap, which is the unoccupied β orbital of the HOMO. For 12-PEDOT, the patterns are similar, but the HOMO–LUMO gap reduces faster for longer chain lengths. For the +2 state, which has triplet ground state, two unoccupied levels appear in the gap; these are the unoccupied β orbitals of the two singly occupied orbitals. The HOMOs are normalized to 0 eV. Without this normalization, the overall energy level scale reduces as electrons are removed.

Figure 7 plots the energy levels for these systems for an energy range around the HOMO–LUMO gap with zero to four anions. For comparison, Figure 8 presents the same energy levels for neutral and oxidised PEDOT without anions. The HOMO–LUMO gap gets progressively smaller as anions are added.

The energy level diagrams corresponding to the PEDOT with and without anions in Figure 7 and Figure 8, respectively, are very similar in character, showing that the oxidation level of the PEDOT rather than the position or presence of anions is the dominant influence on the energy level structure. As seen in other work [6], the states ‘appearing’ in the gap are not new states created by the introduction of anions but occur in PEDOT oxidized both without anions and with anions. In fact, these states are the newly unoccupied HOMO being pushed upwards, whether that is the unoccupied half of a half-occupied HOMO, or a spin-symmetric new LUMO. It is the pushing up in energy of the newly unoccupied levels that causes the bandgap to be reduced.

**Figure 9:**
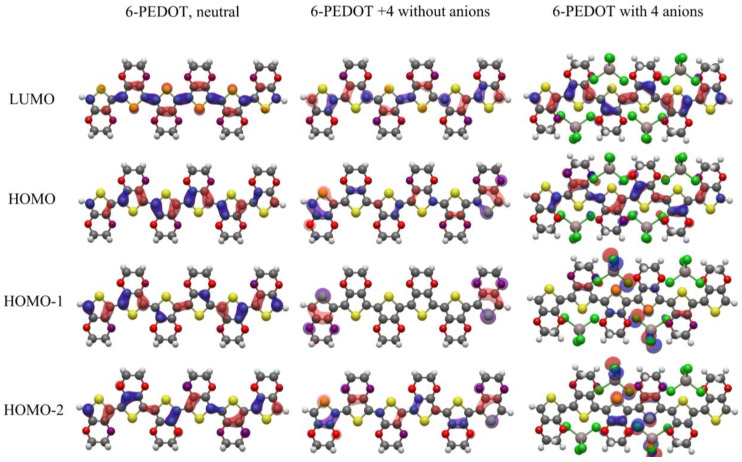
Molecular orbitals, at an isovalue of 0.03, for neutral 6-PEDOT without counterions, 6-PEDOT with +4 oxidation state without counterions and 6-PEDOT with four anions. For the anions, the light grey atoms are aluminium and the green atoms are chlorine. For the PEDOT, the yellow atoms are sulfur, the dark grey atoms are carbon, the red atoms are oxygen and the white atoms are hydrogen. The molecular orbitals show that the HOMO-1 and HOMO-2 for the four-anion case are highly localized on the chlorine atoms of the anions. These highly localized anionic orbitals mix with each other much less than the delocalized orbitals in the system without counterions and are therefore able to bunch more closely in energy than the orbitals in PEDOT without counterions. The HOMO-1 and HOMO-2 of the 6-PEDOT with four anions shows predominantly anionic orbitals with a contribution from the PEDOT orbitals; this is due to a DFT error, and the orbitals should be purely anionic.

A key difference between Figure 7 and Figure 8 is observed at high anion concentrations in Figure 7, where an anion concentration of 0.66 is reached for 6-PEDOT +4. In this case, many energy levels cluster closely under the HOMO. This is caused by Coulombic repulsion between the anions, which causes their orbitals to rise in energy. This result can also be seen in [50], although it is not discussed. These orbitals are highly localized on the chlorine atoms of the anions, as shown in the HOMO to HOMO-2 of 6-PEDOT with four anions in Figure 9. Due to their high localisation, the anionic chlorine orbitals have very similar energies to each other, hence the close stacking of their energy levels in Figure 7. This effect occurs further down the energy scale for 12-PEDOT with anions, because the overall anion concentration is only 0.33 instead of 0.66. This reduces the Coulombic repulsion between the anions and lowers the energy of their orbitals. It is noted that the existence of high levels of Coulombic repulsion is contradictory to the bipolaron model, which predicts that the structural energy savings of co-locating anions could overcome the Coulombic repulsion. Instead, a decrease in anion location stability is observed as discussed in [15].

For 6-PEDOT with four anions, the HOMO-1 and HOMO-2 orbitals have dominant anionic character with a small amount of PEDOT character. The mixing of orbitals is due to a DFT error caused by the self-interaction error corrections. In redox systems, including conducting polymers, the charge transfer is well known to be very close to 1, whereas DFT calculations predict 0.8–0.9. Therefore, it is reasonable to conclude that the HOMO-1 and HOMO-2 are pure anionic orbitals.

### 3.5. Effect of Coulombic Repulsion between Anions on Conductivity

As discussed in the introduction, conductivity begins to decrease at high anion concentrations, after reaching a peak when about half the redox sites are occupied. Figure 9 shows that at anion concentrations of 0.66, localised anionic orbitals take the place of the HOMO-1 and HOMO-2, where at lower anionic concentrations these energy levels were PEDOT orbitals delocalised along the conjugated carbon backbone. The new localised anionic states are too far below the HOMO to interfere with conductivity at room temperature without nucleic motion—the HOMO-1 is around 0.1 eV below the HOMO (Figure 7). However, it is hypothesized that in a macropolymer with thermal nucleic motion, the natural variation in interatomic distances would cause these localised states to be regularly pushed up to a similar or greater energy than the HOMO, where the occupied and unoccupied redox sites that charge carriers hop between are located. This would interfere with the hopping process, lowering conductivity, and would affect both intra- and interchain conductivity. To the best of the authors’ knowledge, this is a novel addition to the theory of conducting polymer conductivity.

### 3.6. Study of the Anion in Isolation

**Figure 10:**
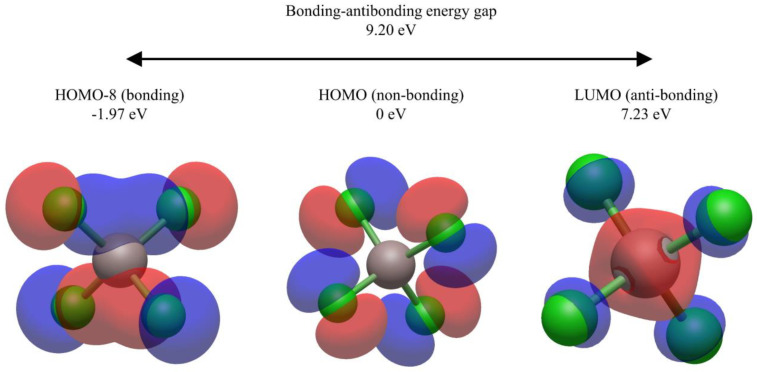
Molecular orbitals at an isovalue of 0.03 for the HOMO–8 (bonding), HOMO (non-bonding) and LUMO (antibonding) of AlCl_4_^−^ with energy levels shown to be normalised to the HOMO of the isolated anion. The non-bonding orbitals are all equal mixtures of the chlorine p-orbitals of similar character to the HOMO, explaining why they sit very closely in energy in the case of 6-PEDOT with four anions in Figure 8.

In order to understand the nature of the states introduced by co-located anions, the anion was studied in isolation. AlCl_4_^−^ has a wide energy gap between its bonding and antibonding states (9.2 eV). When co-located with the PEDOT, the antibonding states sit at high energy, well above the HOMO–LUMO gap of the overall system as shown in Figure 8, while the bonding states sit at low energy, well below the gap. When the anion is considered in isolation, there are eight p-derived non-bonding states on the AlCl_4_^−^ anion from HOMO to HOMO-7. The bonding, non-bonding and antibonding states are shown in Figure 9. All of the non-bonding states are chlorine p-orbitals of similar character to the anionic HOMO. It is clear from comparing Figure 9 and Figure 10 that the anionic orbitals appearing in the HOMO-1 and HOMO-2 for 6-PEDOT with four anions have the character of the anionic non-bonding orbitals. None of the anionic orbitals contribute to unoccupied energy levels appearing in the gap as the number of anions increases, in contrast to the predictions of the bipolaron model [29].

## 4. Conclusions

The results and analysis presented here add further evidence to move forwards from the original bipolaron model developed from pre-DFT methods. In PEDOT with AlCl_4_^−^ anions, distortions localise around the anions, and when multiple anions are located near each other, a single longer distortion appears across them. However, there is no driving force for anions to co-locate. The anions have Coulombic repulsion between them and will displace each other if concentration levels are too high [15]. The lowest energy configurations have anions well distributed along the oligomer and not too close to the ends.

Two distinct distortion characteristics have been observed in this work, identified by a single or a double peak in quinoid structure. It is noted that other numbers of distortion peaks and different distortion shapes are possible on longer chains with different anion configurations, with the bond length alternations correlated to the charge density in the PEDOT. The original bipolaron model is based on similar descriptions of distortions and uses the terms polarons, bipolarons or polaron pairs. However, the distortions observed in this work are not reliably associated with +1 or +2 states, as predicted by any version of the bipolaron model. Instead, the distortions shapes are dependent on many aspects including oxidation state and configuration of anions, oligomer length, anion type and functional. For PEDOT with anions, higher local anion concentrations are associated with double peak distortions appearing as the ground state. The double peak distortion may be similar to the distortion type observed experimentally for longer polythiophene oligomers without counterions with +2 oxidation state [47].

Some have begun to adapt the terminology of the bipolaron model to reflect the modern understanding [6,50]. Consequently, a more precise definition of polarons and bipolarons is called for, as already partially explained by these authors. A polaron is a localised, singly charged state, caused in experiment by the introduction of an anion, and almost impossible to introduce in real life without an anion present. A polaron appears as a spin on an ESR measurement due to having a half-occupied HOMO. A bipolaron is what happens when two anions adsorb near enough to each other that the polarons overlap and the original HOMO local to the anions becomes fully emptied. This causes the ESR spins to disappear. This pattern repeats as increasing odd and even numbers of anions are introduced near to each other. Consequently, the main difference between polarons and bipolarons is that polarons are a localized state with spin asymmetry while bipolarons are a localised state having spin symmetry. Apart from that, there are no further differences between polarons and bipolarons, and no definite limit on the number of anions that can share a single distortion.

The even distribution of anions throughout the polymer to begin with means that ESR spins build up during charging, similar to the concept of the polaron pair as proposed by Sahalianov et al. [50]. Then, as the anion concentration rises, the anions are forced to locate nearer to each other, so that the polarons overlap and the half-occupied levels become unoccupied and the spins disappear. While each anion introduces one bonding and one antibonding state, these are far below and above the HOMO–LUMO gap, respectively. The apparent new state appearing in the HOMO–LUMO gap is the newly unoccupied half or whole delocalised PEDOT energy level which has been pushed up by the occupied levels below it into the gap, with the non-bonding anionic orbitals clustering below the HOMO.

At the highest anion concentration modelled at 0.66, the non-bonding orbitals of the anions are promoted to an energy level approaching that of the system HOMO, driven by Coulombic repulsion between the anions. It is hypothesized that in the macropolymer, these highly localised states would be regularly promoted to the energy of the sites responsible for charge carrier hopping as a result of nucleic motion, interfering with both intra- and interchain conductivity. The proposed interference with the hopping mechanism(s) is hypothesized to contribute to, or cause, the loss of conductivity found in conducting polymers at anion concentrations above 0.5. This hypothesis is a novel contribution to conducting polymer theory.

This work naturally leads towards further investigation of the thermodynamics of the PEDOT/AlCl_4_^−^ system. The system demonstrates clear anion site stability, which implies a resistance to facile reorganization of anions and may explain the sloping charge/discharge curve seen in experiment [9]. In other words, the pairing may be more suitable to pseudocapacitive storage than battery-like storage, in that a surface redox effect is more achievable than diffusing the anions deep into the polymer due to lack of ease of anions migrating through the bulk of the material as is seen for Al–graphite, which has a very flat charge/discharge curve due to the nearly equivalent energies of the anions in different positions in graphite [74]. A more detailed study of the thermodynamics of the system could predict a chemical potential/anion concentration curve, which might help explain the experimental results.

## Data Availability

All data supporting this work can be found in the file Single Chains Comparison.xlsx within the folder Post-processing_spreadsheets.zip at https://doi.org/10.5258/SOTON/D2915.

## Supplementary Materials

The following supporting information can be downloaded at: https://www.mdpi.com/article/10.3390/polym16101376/s1. Figure S1. the relaxed configurations of the PEDOT oligomers with anions for which the energy levels are presented in Figure 8. Anions are placed either above or below the oligomer, which can be observed via whether the PEDOT or the anion appears ‘in front’ in the case of overlaps between atoms in the molecular drawings.
