# Supplementary material for: An Electronic Structure Investigation of PEDOT with AlCl4− Anions—A Promising Redox Combination for Energy Storage Applications
Source: Polymers (Basel). 2024 May 11;16(10):1376. doi: 10.3390/polym16101376 (PMC11125351; doi:10.3390/polym16101376)
Supplement: Supplementary file 1 [file polymers-16-01376-s001.zip › polymers-2947974-supplementary.pdf]

polymers

MDPI

Supplementary Material

An Electronic Structure Investigation of PEDOT with $AlCl_4^-$ Anions—A Promising Redox Combination for Energy Storage Applications

Ben Craig, Peter Townsend, Chris Kriton-Skylaris, Carlos Ponce de Leon and Denis Kramer

The energy level structure in Figure 8 was based on 6- and 12-PEDOT with anions. The following table shows where the anions are located on the oligomers. These are the lowest energy states found for that number of anions from either a complete sample for 6-PEDOT or large random sample for 12-PEDOT.

Copyright: © 2024 by the authors. Licensee MDPI, Basel, Switzerland. This article is an open access article distributed under the terms and conditions of the Creative Commons Attribution (CC BY) license (https://creativecommons.org/licenses/by/4.0/).

Number of anions	6-PEDOT	12-PEDOT
1		
2		
3		
4		

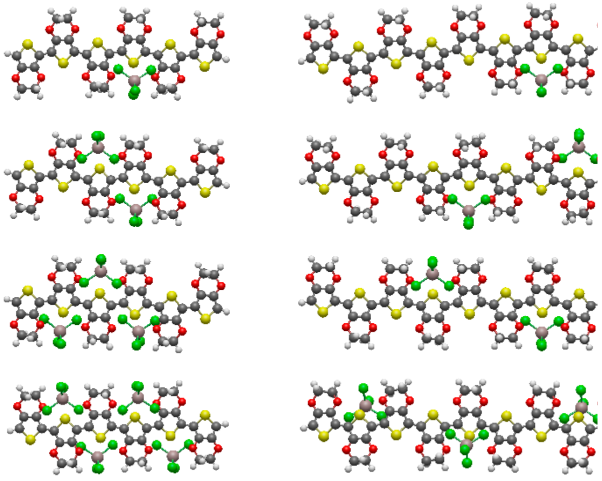

Figure S1. the relaxed configurations of the PEDOT oligomers with anions for which the energy levels are presented in Figure 8. Anions are placed either above or below the oligomer, which can be observed via overlaps in the molecular drawings.
